# Assessment of the Need for Designing an Interactive Educational Tool on Exophytic Lesions of the Oral Mucosa

**DOI:** 10.7759/cureus.69867

**Published:** 2024-09-21

**Authors:** Pegah Mosannen Mozafari, Seyedeh Soudabeh Fatemi, Zahra Delavarian, Majid Akbari, Mohsen Gholizadeh, Mozhgan Kazemian, Parham Aghasizadeh Sharbaf, Seyed MohammadReza Aboutorabzadeh

**Affiliations:** 1 Oral and Maxillofacial Diseases Research Center, Mashhad University of Medical Sciences, Mashhad, IRN; 2 Oral Medicine, Mashhad University of Medical Sciences, Mashhad, IRN; 3 Restorative Dentistry, Mashhad University of Medical Sciences, Mashhad, IRN; 4 Faculty of Medicine and Dentistry, University of Alberta, Alberta, CAN; 5 Oral and Maxillofacial Surgery, Mashhad University of Medical Sciences, Mashhad, IRN; 6 Biotechnology, Kavian Institute of Higher Education, Mashhad, IRN; 7 Research, Shahab Oral Medicine and Special Care Dental Clinic, Mashhad, IRN

**Keywords:** dental students learning, electronic courseware, electronic learning, interactive educational product, needs assessment, oral mucosal exophytic lesions

## Abstract

Introduction

This study sought to evaluate the necessity of creating an interactive educational resource for instructing dental students on oral exophytic lesions. It also aimed to determine the validity and reliability of a questionnaire designed to assess the quality of educational software tailored to these lesions.

Materials and methods

This descriptive cross-sectional study involved 102 dental students from Mashhad University of Medical Sciences, Iran, who had completed the theoretical course on oral exophytic lesions. A paper questionnaire, including 23 items, assessed their clinical knowledge, the efficacy and limitations of existing educational resources, and the need for interactive electronic courseware (e-courseware). The questionnaire's validity and reliability were evaluated through expert feedback and statistical measures (intraclass correlation coefficient (ICC) and Cronbach's alpha).

Results

Students' answers to the seven items about evaluating their clinical diagnosis knowledge of oral exophytic lesions were between four and five items correct on average. This finding indicated that students' overall skill in diagnosing exophytic lesions was poor to moderate. The study highlighted a moderate level of self-assessed clinical ability in diagnosing oral exophytic lesions among students (62.7%), with a preference for textbooks and color atlases as primary information sources. The disadvantages of existing electronic resources were noted, alongside a strong student consensus (88.23%) on the need for interactive e-courseware featuring comprehensive, visually engaging content for differential diagnosis education. The reliability and validity analyses of the questionnaire underscored its appropriateness for assessing educational needs. In this regard, ICC for the usability of scientific context, training ability, and interaction was 0.92, 0.73, and 0.82, respectively. Also, Cronbach's alpha score was at 0.90.

Conclusion

The research underscores a significant gap in dental students' knowledge and diagnostic skills regarding oral exophytic lesions. It emphasizes the critical need for an innovative, interactive educational tool that aligns with contemporary students' digital learning preferences. The envisioned e-courseware would facilitate self-learning and address current resources' limitations, potentially transforming dental education by enhancing clinical diagnostic skills through accessible, effective, and engaging digital content.

## Introduction

Today, virtual education using educational software takes pivotal steps to advance education systems. Utilizing usual instruction methods in classrooms decreases due to the limitations of place and time and the restriction of printed academic texts [1]. Interestingly, the COVID-19 pandemic revealed that using new technologies for educational purposes can be a practical alternative approach to instruction anywhere at any time [2]. Therefore, electronic media and other communication technologies are preferred for use in new teaching methods [3]. In this regard, interactive electronic programs have caused the advancement of computer-based learning tools over the years. The learning with this system is performed by presenting the combination of practical principles and digital images with theoretical information texts attached to clinical samples [4,5]. However, in many countries, most electronic and computer clinical diagnosis tools are limited to pictures and electronic textbooks (e-textbooks), and for teaching clinical practices, the educational methods remain traditional. Instructional reformations in this field can decrease the presence in attendance classes and create interactive applications from a distance [6].

The prevalence of oral exophytic lesions, defined as growing pathologic projections above the normal contours of the oral mucosa, is increasing. Because of the abundant diversity of these lesions, it could be possible that they were mistakenly considered clinically similar with distinct histopathologic characteristics. As a result, a precise diagnosis of them is essential [7]. According to the importance of accurate diagnosis of these lesions and distinguishing benign and malignant entities, educating oral exophytic lesions with electronic products can be effective in dental students' clinical skills learning procedures like similar studies [3,4,8].

Since dentists' level of knowledge and diagnosis skills about the different types of mucosal lesions are prerequisites for the acceptance of treatment responsibility, any investigation causing the identification of these requirements can influence their scientific and practical knowledge. Also, it can be helpful to recognize the current dental education system and assist in proper instruction planning for preventing and treating oral diseases. Hence, dental students' oral exophytic lesions diagnosis ability should be evaluated for needs assessment of developing a new teaching tool on this subject.

To our knowledge, no e-courseware currently exists for the education of oral exophytic lesions; consequently, no needs assessment has been conducted to date. This study aimed to assess the need to design an interactive educational tool regarding oral exophytic lesions. Besides, the validity and reliability of the questionnaire on the quality of the educational software on these lesions were evaluated.

## Materials and methods

In this descriptive cross-sectional study, to assess the need to design an interactive educational software on oral exophytic lesions, 102 dental students in the first semester of the 2018-2019 academic year at the Faculty of Dentistry, Mashhad University of Medical Sciences, Mashhad, Iran, who passed the theoretical course of the oral exophytic lesions, were voluntarily included.

Initially, a paper questionnaire was distributed to evaluate the level of clinical knowledge and diagnosis ability of oral exophytic lesions in studied students and the need to design an educational aid product. The questionnaire assessed the needs assessment by 23 items, consisting of clinical skills, information resources used by the student to enhance diagnostic skills, disadvantages of electronic resources, and students' willingness to design an e-textbook (Table 1).

**Table 1:** Translated needs assessment questionnaire from Persian

Question items	Options
Sex	Female
	Male
(1) What is your evaluation of your clinical ability to diagnose and manage oral mucosal exophytic lesions?	(A) Poor
	(B) Minimal
	(C) Moderate
	(D) Good
	(E) Excellent
(2) Which one of these lesions can commonly appear as an exophytic lesion in the mouth?	(A) Leukoplakia
	(B) Peripheral odontogenic tumors
	(C) Hard tissue tumors
	(D) Salivary gland tumors
	(E) Hamartomas like hemangioma
	(F) Inflammatory hyperplasia
	(G) Pemphigus
	(H) Soft tissue malignancies
(3) Which one of these lesions can occur ulceratively in children without any evidence of irritation?	(A) Inflammatory hyperplasia
	(B) Fibrosarcoma
	(C) Lipoma
	(D) Benign salivary gland tumor
	(E) Mucocele
	(F) Ranula
(4) Which one of these mucosal lesions can be observed as an exophytic lesion with a smooth surface and the same color as the oral mucosa?	(A) Squamous cell carcinoma
	(B) Verrucous carcinoma
	(C) Papilloma
	(D) Salivary gland tumor
(5) For which one of these exophytic lesions the observation of radiologic changes in the jaw is necessary to diagnose it?	(A) Salivary gland tumor
	(B) Osteosarcoma
	(C) Squamous cell carcinoma
	(D) Inflammatory hyperplasia
(6) Which one of these exophytic lesions can commonly be pedunculated with the appearance of a prominent nodule hanging from the oral mucosa?	(A) Squamous cell carcinoma
	(B) Salivary gland tumor
	(C) Papilloma
	(D) Inflammatory hyperplasia
(7) Which one of these exophytic lesions is susceptible to bleeding by slight irritations?	(A) Pyogenic granuloma
	(B) Malignant minor salivary gland tumor
	(C) Capillary hemangioma
	(D) Nevus flammeus
(8) For diagnosis of which one of these lesions the mobility of the adjacent tooth in an exophytic gingival lesion is necessary?	(A) Squamous cell carcinoma
	(B) Inflammatory hyperplasia
	(C) Melanoma
	(D) Osteosarcoma
(9) What information resources do you use to improve your diagnosis ability of exophytic lesions? (You can choose several options.)	(A) Textbooks
	(B) Color atlases in the university library
	(C) Internet
	(D) Oral disease specialized websites. Name of the site: ________
	(E) Other resources (Please mention them: _____________)
(10) How much do you think demonstrated seminars in the department can improve your diagnosis ability of exophytic lesions?	(A) They are sufficient 100%
	(B) They are almost sufficient, but using other resources is required
	(C) They supply low scientific needs, and using other resources is strongly felt
	(D) They are insufficient, and only by studying other resources can diagnosis abilities be completed
(11) How much do you think examining patients with exophytic lesions in the oral disease clinical course can increase your knowledge?	(A) It can sufficiently increase our knowledge about all exophytic lesions
	(B) It is only helpful for the examined lesions, and studying is required for other lesions
	(C) Although it can increase our experience of diagnosing the examined lesions, studying other resources is required for differential diagnosis
	(D) Only studying other resources can increase our knowledge, and patients' examinations cannot do it
(12) If you want to acquire clinical exophytic lesions' diagnosis ability, which one is your choice as a resource? (You can choose several options.)	(A) Textbooks
	(B) Class handouts
	(C) Color atlases in the university library
	(D) Electronic resources
(13) In your opinion, which is more available, helpful, and practical for the differential diagnosis of exophytic lesions?	(A) Textbooks
	(B) Class handouts
	(C) Color atlases in the university library
	(D) Electronic resources
(14) In your opinion, which is the best in terms of comprehensiveness, colorful images, and comparative information to improve your clinical skills in the differential diagnosis of exophytic lesions?	(A) There are no comprehensive and colorful resources detailing differential diagnoses
	(B) The existing electronic resources are thorough and entirely meet our need to diagnose the lesions
	(C) Textbooks entirely meet our need to diagnose the lesions
	(D) The color atlases in the university library are enough for every dental student and entirely meet our need to diagnose the lesions
	(E) Both textbooks and color atlases together
(15) In your opinion, what are the most important disadvantages of existing electronic resources for oral exophytic lesions' differential diagnosis? (You can choose and write several options.)	(A) Being English
	(B) The possibility of not having documented scientific and practical information on the websites
	(C) Lack of familiarity with relevant and reliable websites
	(D) Requires special software to use existing websites
	(E) The possibility of being different in educational method compared to the Iranian standard educational method
	(F) For using some websites, paying limited membership charges is required
	(G) Write other disadvantages you think: ________________
(16) If an electronic textbook (e-textbook) with colorful images of common kinds of oral mucosal exophytic lesions alongside their differential diagnoses is produced and uploaded on the university website by the oral medicine department's professors, how much can it supply your needs in exophytic lesions' differential diagnosis?	(A) It can be excellent
	(B) It can be helpful
	(C) It does not differ from the current situation
	(D) It can be more confusing for students
(17) Do you agree with presenting academic seminars about exophytic lesions' differential diagnosis in a virtual textbook and you only examine the patients in practical courses?	(A) Strongly agree
	(B) Agree
	(C) Neither agree nor disagree
	(D) Disagree
	(E) Strongly disagree
(18) Do you agree with considering a part of your practical grade for oral exophytic lesions according to reviewing the virtual textbook and answering its questions?	(A) Strongly agree
	(B) Agree
	(C) Neither agree nor disagree
	(D) Disagree
	(E) Strongly disagree
(19) Have you ever read the university website's e-textbook on oral white and red lesions? If your answer is "Yes," in your opinion, how much could this e-textbook help improve your clinical skills?	(A) Yes
	(B) No
	(A) Much more than acceptable
	(B) Above average
	(C) Average
	(D) Less than acceptable
	(E) Unacceptable
(20) Since several virtual textbooks in different dentistry fields were uploaded to the university website, which of them have you read yet? Please mention them.	___________________________________
(21) If you have not read them yet, what is your cause?	(A) The lack of providing information by the professors
	(B) Problems in the registration procedure
	(C) The website's traffic
	(D) Uninterested in using the Internet and electronic resources
	(E) The lack of availability of the Internet
	(F) The e-textbooks cannot answer our needs
	(G) The e-textbooks focus on unimportant matters
	(H) I achieve good clinical skills that do not need a textbook
	(I) Other causes (Please mention them: _____________)
(22) In your opinion, what is the most helpful format for presenting e-textbooks?	(A) Case and problem-based
	(B) Review of theory subjects in the form of a summary
	(C) Additional subjects that are not demonstrated in the classes
	(D) Case and problem-based alongside multiple-choice questions and their feedback
(23) A: How much has your differential diagnosis ability improved in the department's seminars for exophytic lesions?	A: __________________________________
B: Can you mention some Persian websites that educate on exophytic lesions' differential diagnosis? (If possible, please write their addresses.)	B: __________________________________

The first item of the questionnaire asked students to evaluate their clinical skills. Also, items 2 up to 8 contained multiple-choice clinical questions about oral exophytic lesions to assess dental students' clinical knowledge. For grading the level of their clinical knowledge about these lesions, three levels were considered, which were poor (0-3 correct answers), average (4-5 correct answers), and good (6-7 correct answers).

Moreover, items 9-14 inquired about students' information resources to improve their diagnosis ability of oral exophytic lesions and the value of the oral and maxillofacial department's seminars and patients' examinations. The disadvantages of available electronic resources for the differential diagnosis of oral lesions were questioned in item 15. Besides, items 16 up to 24 assessed dental students' willingness to use electronic and virtual textbooks in different fields and their reasons.

To evaluate the questionnaire's validity, eight Oral and Maxillofacial Disease Department professors of the Faculty of Dentistry at Mashhad University of Medical Sciences appraised it. Then their collected opinions and comments were performed on the questionnaire.

Furthermore, the questionnaire's internal consistency reliability was measured with the intraclass correlation coefficient (ICC) and Cronbach's alpha score. In this regard, for test-retest reliability of the designed questionnaire, 13 dental students eligible for this study with simple random sampling using the website http://www.randomizer.org were chosen and answered twice to the questionnaire within two-week intervals. The numbers 12, 40, 23, 2, 73, 96, 101, 45, 32, 81, 65, 6, and 56 were picked from the Faculty of Dentistry classes lists.

Finally, the descriptive statistical analyses were performed via IBM SPSS Statistics for Windows, Version 22.0 (Released 2013; IBM Corp., Armonk, New York, United States).

## Results

**Figure 1:**
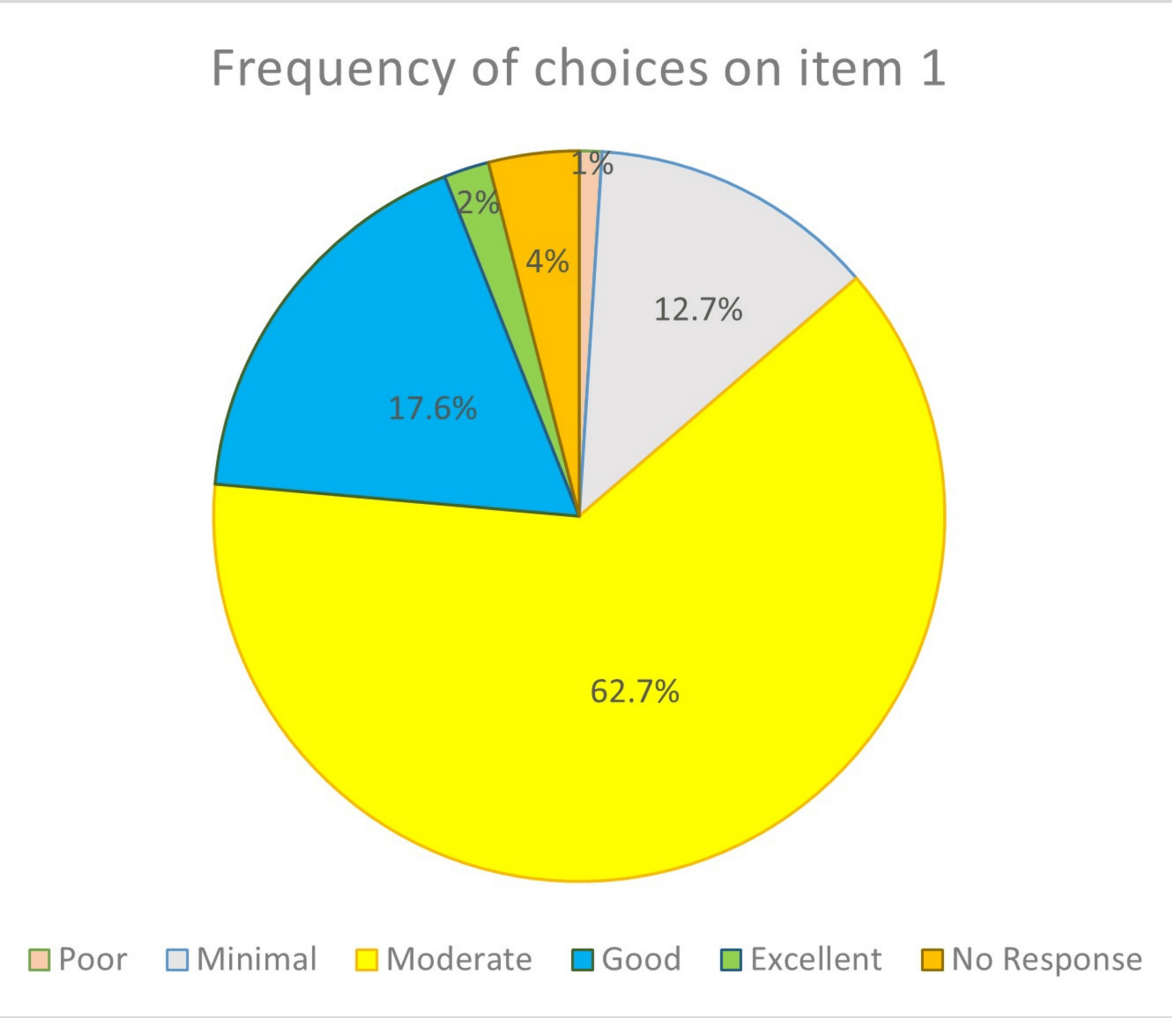
Dental students' evaluation of their clinical ability to diagnose and manage oral exophytic lesions

The participants of this study were 76 females (74.5%) and 26 males (25.5%). The most studied dental students (62.7%) evaluated their clinical ability to diagnose and manage oral exophytic lesions as moderate in the first item of the questionnaire (Figure 1).

Also, their answers to the second to eighth items about assessing their clinical knowledge of this field were between five and five items correct on average (average level of clinical knowledge).

**Figure 2:**
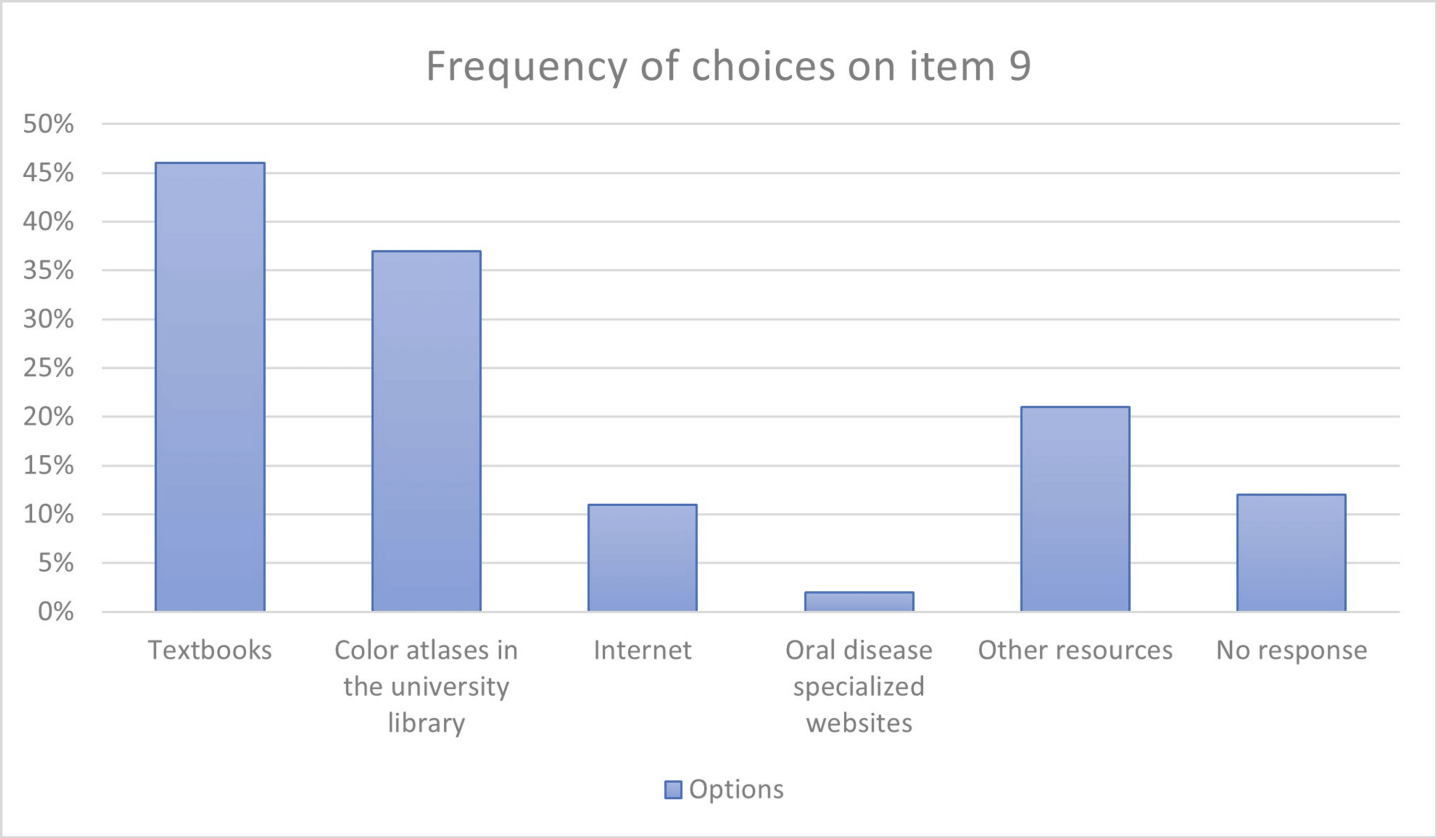
Dental students' information resources for improving their diagnosis ability of the lesions

The outcomes of students' information resources for improving their diagnosis ability of the lesions demonstrated that most of them used textbooks and available color atlases (Figure 2).

In addition, their belief in the sufficiency of the department's seminars showed that 3.92% of students believed the seminars were 100% sufficient and 47.05% chose the option describing the seminars almost improved their diagnosis ability of exophytic lesions in the 10th item. However, 40.19% and 2.94% of participants thought the seminars' efficiency was low and insufficient, and 5.88% did not answer this item.

The findings from practical experiences and patient examinations showed that while half of the students believe that practical courses involving real lesions can enhance their diagnostic skills, they also recognize the necessity of consulting additional resources for differential diagnosis and enhancing their clinical expertise. Although 7.84% of this item of the questionnaire remained without an answer, other responses included 9.80% of dental students knew these experiences sufficiently, 30.40% chose the option defining it is only helpful for the examined lesions and studying is required for other lesions, and 1.96% thought they are entirely insufficient.

**Figure 3:**
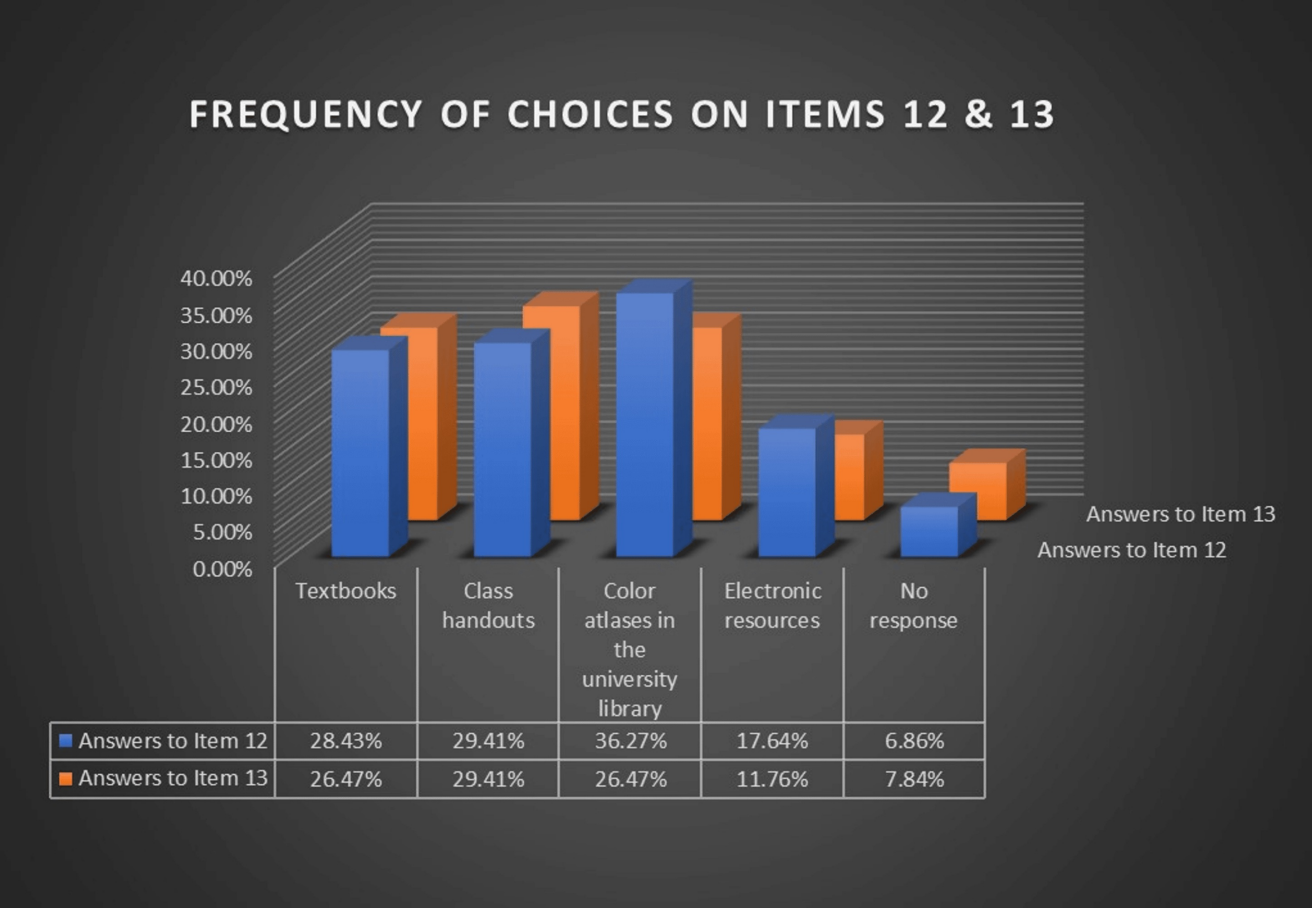
Dental students' chosen resources for acquiring clinical diagnosis ability of the lesions in terms of the current students' situation and practicability (items 12 and 13)

Although the answers on item 12 showed that the most chosen resource (36.27%) for acquiring clinical exophytic lesions' diagnosis ability was color atlases in the university library, most students (29.41%) believed class handouts were more available, helpful, and practical for exophytic lesions' differential diagnosis on item 13 (Figure 3).

The studied students' opinions about the best source to improve their clinical skills in exophytic lesions' differential diagnosis in terms of comprehensiveness, colorful images, and comparative information, questioned in item 14, demonstrated that 28.43% of students expressed the colorful atlases in the university library are enough and both textbooks and electronic resources were chosen equally (17.64%). Also, 27.45% asserted that no comprehensive and colorful resources exist for this purpose, and 8.82 left the question without an answer.

Findings presented that the most crucial disadvantage of available electronic resources for oral exophytic lesions' differential diagnosis in the studied students' opinion (39.41%) was the need for special software. Other negative points were the possibility of using different methods compared to the Iranian standard educational process (22.54%), the obligation of paying a membership fee (19.60%), the probability of the websites' information issues (16.66%), inability to understand English language (12.74%), and unfamiliarity with relevant and reliable websites (10.78%). Additionally, in 9.80 of cases, the question remained without response.

Most students (88.23%) declared that producing an e-textbook with colorful images of common kinds of oral mucosal exophytic lesions alongside their differential diagnoses by the department would be excellent and helpful. In addition, 3.92% believed this production could be confusing, and 7.84% did not respond to item 16.

**Figure 4:**
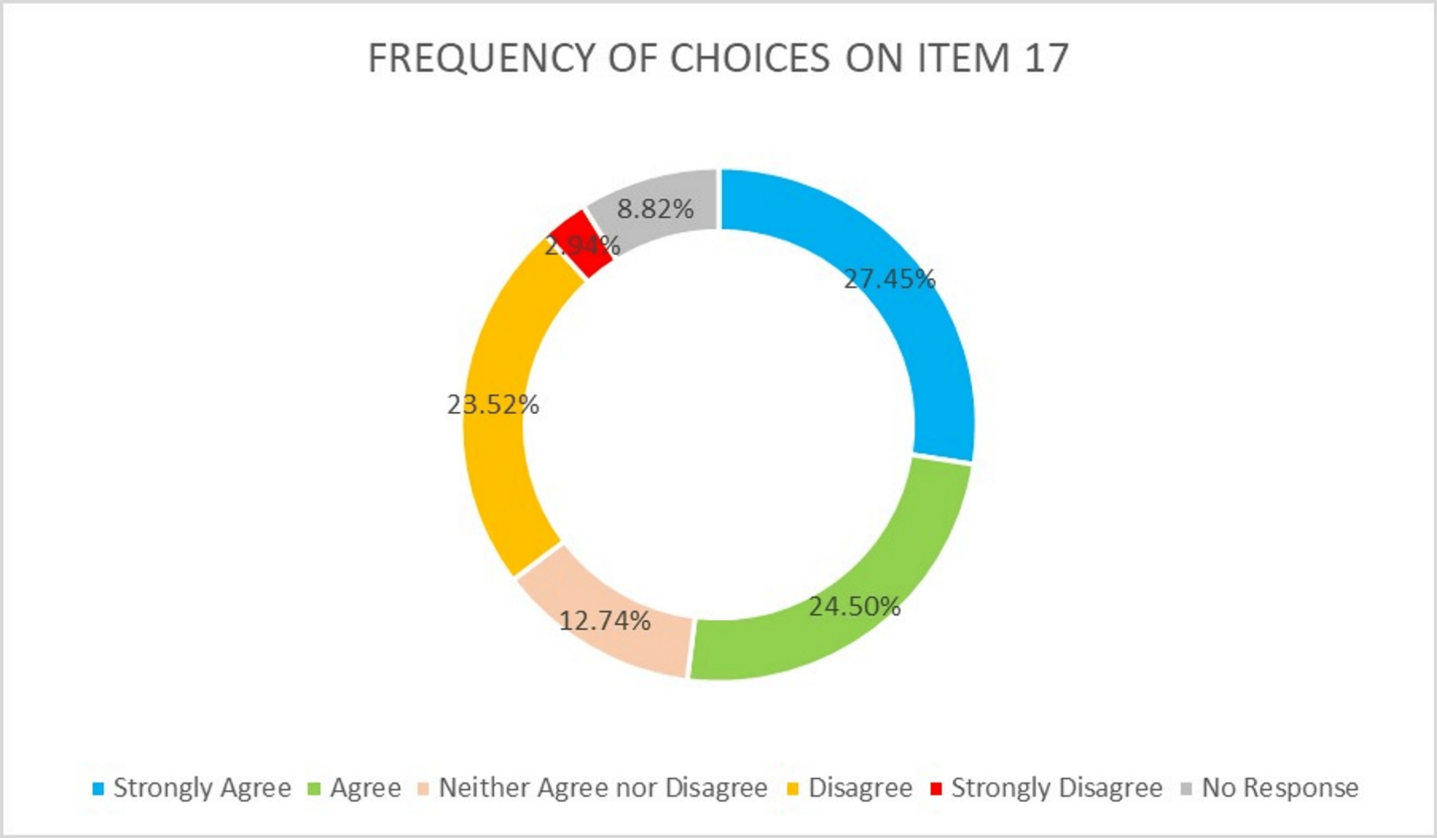
Dental students' agreement on the production of a virtual textbook presenting exophytic lesions' differential diagnosis seminars for part of their practical courses

Outcomes on item 17 presented that most students chose strongly agree (27.45%) or agree (24.50%) with exophytic lesions' differential diagnosis presentation seminars will be provided in a virtual textbook and their practical courses only focused on the patient's examination (Figure 4).

Moreover, half of the studied dental students agreed that a part of their practical grade for oral exophytic lesions could be considered according to reviewing the virtual textbook and answering its questions. On the other hand, a quarter of students opposed this suggestion, and 8.82% of them left this item (item 18) without an answer.

**Figure 5:**
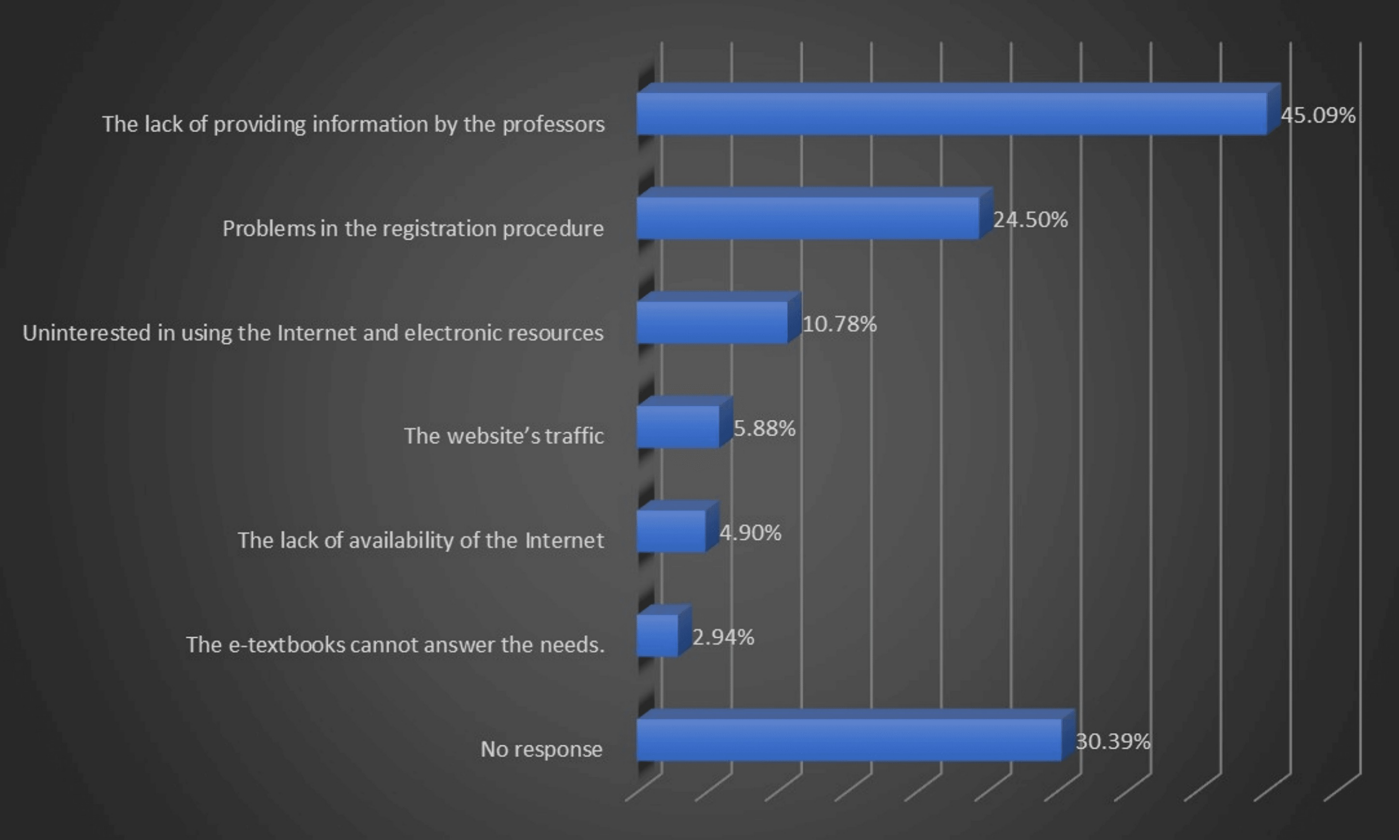
Dental students' causes for not using the university's e-courseware

Only one dental student had read the university website's e-courseware about oral white and red lesions and evaluated it as above average for improving clinical skills. Two students mentioned they had read the endodontic e-textbook, and one subject indicated reading the pathology e-textbook on the university website. Figure 5 exhibits that the chosen options by students were the causes of not reading the university's e-textbooks (Figure 5).

Item 22 findings showed that most preferences of studied dental students for designing the format of the proposed e-textbook were the case and problem-based (45.09%) and case and problem-based alongside multiple-choice questions and their feedback (29.41%) formats, respectively. In 19.60% of cases, this item remained unanswered, 2.94% preferred a review of theory subjects in the form of a summary in the e-textbook, and 1.96% wanted a format with additional subjects that were not demonstrated in the classes. Furthermore, the last item in the questionnaire was not answered by any student.

The questionnaire's internal consistency reliability results demonstrated that ICC for the usability of scientific context, training ability, and interaction was measured at 0.92, 0.73, and 0.82, respectively. Also, Cronbach's alpha score was calculated at 0.90.

## Discussion

Using novel technology-based tools is prominently increasing globally, causing a rise in information availability and leading to the improvement of self-learning [9]. Dental and medical educational systems development can be reached by utilizing educational software or interactive tools as powerful multimedia devices with a combination of visual and auditory data [10]. In this regard, the efficacy of interactive tools has been reported in several studies [5,9,11-13].

Based on the wide variety of oral exophytic lesions, it is not possible for dentistry students to have the chance to experience all of these lesions during their limited education schedule. On the other hand, regarding the limited number of oral medicine specialists and lack of patient information about oral lesions in Iran, it is highly probable that general dentists face these patients in everyday practice. As a result, it is essential that oral medicine education for undergraduate dental students be emphasized to lead to a precise diagnosis or at least proper referral of patients to particular centers. The current study evaluated the needs assessment of designing an interactive educational tool on oral exophytic lesions for dental students at Mashhad University of Medical Sciences. The outcomes presented that students' diagnosis ability was not high enough, which noticed a lack of an educational complementary tool for the instruction of these lesions. In this regard, most studied students agreed with introducing interactive software for improving their oral exophytic lesions' diagnosis ability.

The findings of two studies in Iran demonstrated that the ability of general dentists to diagnose oral lesions was less than 50% [14,15]. Moreover, a study in Brazil reported that dentists' knowledge about oral lesions was less than expected [16]. These studies presented that oral exophytic lesions encompass a wide range. However, students in the oral medicine department, limited by their brief exposure during semesters of practical oral medicine courses, often miss the opportunity to encounter all such lesions firsthand. The rarity of some lesions only exacerbates this gap in experience. However, the infrequency of these conditions does not diminish their importance; overlooking such lesions can adversely affect patient outcomes and survival rates. In a competent general dental practice, it is crucial for dentists to identify lesions accurately or to determine the necessity of referral to a specialized tertiary care center for potentially life-threatening conditions. Developing interactive software that challenges students to learn about various oral lesions could significantly enhance their diagnostic skills and knowledge.

Basirat et al. evaluated the oral lesion diagnostic skills of senior dental students at another university in Iran, and their outcomes showed that students believed their ability was average. Also, they tended to have an additional supplementary course on prevalent oral lesions in their curriculum [17].

In the Sarabadani et al. study, most dentists believed that they required oral medicine continuing medical education (CME) courses even though 79% of them were not inclined to participate in CME courses [14]. This surprising finding might be associated with the lack of these programs' efficacy, being time-consuming, and interfering with dentists' careers. Therefore, creating an e-courseware that leverages new technologies without impeding practice time or requiring relocation could significantly enhance the ability of dental students and general dentists to diagnose oral lesions.

Similar to the present study, a survey about students' perceptions of online distance learning presented that most students preferred this new method compared to the traditional teaching approach because online education can provide a facility to remove educational obstacles, such as time, heavy workload, and domestic problems [18]. In addition, the advantage of virtual education over the traditional way of endodontic problem-solving was proposed in some studies [13,19]. In addition to the benefits mentioned, the opportunity for repeated exposure to teaching materials and the trend among the new generation of dentists who are familiar with and inclined to use electronic resources underscores the importance of developing new e-courseware for future dentistry learners.

The complexity of a precise differential diagnosis in the oral medicine field and dental students' weakness in oral lesion diagnosis can be good reasons to propose using unprecedented teaching techniques, like related software, to boost dental students' clinical skills [20]. Despite the importance of using diverse methods to instruct oral medicine lessons, very few new technological pathways were introduced for this purpose, particularly in Iranian dental schools [14].

Assessing students' feedback on the substitution of computer or mobile-based tools over traditional educational approaches and emphasizing this necessity can support and accelerate the usage of these devices. The current study's findings highlighted the value of decreasing education times in traditional classrooms for increasing online and distance teaching methods. In this regard, further investigations are recommended to evaluate the efficacy of interactive educational tools based on novel technologies in improving dental students' oral exophytic lesion diagnosis. The authors of this study tried to use the present outcomes for designing and using an interactive educational tool in another survey.

## Conclusions

It seems to be true that the outcomes of the needs assessment questionnaire for evaluating the diagnosis ability of oral exophytic lesions in the dental students of Mashhad University of Medical Sciences showed insufficient and deficient knowledge. As a result, developing and introducing an interactive educational tool to teach these oral mucosal exophytic lesions is considered essential. This tool can be designed with some specific traits, such as introducing it in the regional language, being free of charge, being non-mandatory, and without needing registration procedures.
